# A novel approach for DNA extraction of white spot syndrome virus detection in penaeid shrimp

**DOI:** 10.1038/s41598-025-98536-5

**Published:** 2025-05-05

**Authors:** Vernita Priya, Raja Sudhakaran

**Affiliations:** 1 Aquaculture Biotechnology Laboratory, School of Biosciences and Technology, Vellore Institute of Technology, Vellore, 632014 Tamil Nadu India; 2 Department of Integrative Biology, School of Bio Sciences and Technology, Vellore Institute of Technology, Vellore, 632014 Tamil Nadu India

**Keywords:** DNA extraction, White spot syndrome virus, Shrimp pathogen, PCR, Copy number, Biotechnology, Molecular biology, Zoology

## Abstract

Penaeid shrimp and other aquatic crustaceans are highly susceptible to infection by the White Spot Syndrome Virus (WSSV). In recent years, genomic DNA extraction techniques have become central to many molecular diagnostics and technologies. DNA extraction kits were not included in the comparison because they often lack the flexibility and cost-effectiveness needed for large-scale applications in aquaculture research. This study introduces a novel Dimethyl Sulfoxide (DMSO)-based DNA extraction method that is simple, rapid, and equally sensitive, marking a unique advancement in aquaculture research. We used identical sample volumes to compare the DMSO-based method with the traditional phenol-chloroform and Guanidium Hydrochloride techniques. In the study, the DNA yield obtained using DMSO was 378.4 ng/µL, which was significantly higher compared to the other two extraction methods. The phenol-chloroform method yielded 267.8 ng/µL, while the GHCL method produced 151.2 ng/µL. The DMSO method has a lower detection limit and can detect DNA at concentrations as low as 10⁻⁹, whereas the GHCL and Phenol-Chloroform methods have detection limits of 10⁻⁷. These results suggest that DMSO provides a higher DNA yield and has a lower detection limit than the phenol-chloroform and GHCL methods, indicating its potential for more efficient DNA extraction. Therefore, our study introduces a high-quality genomic DNA extraction protocol applicable to the diagnosis of diseases in other marine organisms.

## Introduction

Marine species constitute the main source of animal proteins that feed humans, making them crucial to the world’s food supply security^1^. Aquaculture with genomic assistance can boost yield while protecting the environment, ensuring enough and sustainable production for global food security^2^. DNA extraction is a fundamental technique in molecular biology for isolating and purifying genomic DNA from biological samples for various downstream applications. Numerous methods can be used to extract DNA from various biological sources; however, recovering high-quality DNA is a difficult step for further use, including electrophoresis, polymerase chain reaction (PCR), and sequencing^1^. The degree to which unaffected and adequate DNA may be isolated from samples varies depending on the tissue’s physical-biochemical characteristics^3^. Thus, developing sensitive, time-efficient, and cost-effective methods is important. Most modern procedures, including genetic mapping and fingerprinting, require pure and quick DNA extraction^4^. PCR-based techniques for studying genomic DNA are becoming increasingly popular both in research and for everyday reasons. An effective DNA isolation approach is required to maximize the potential of PCR technology^5^. The extraction of biomolecules, such as DNA, RNA, and protein, is essential for downstream processes and product development such as diagnostic kits. It can be isolated from any biological material for analytical or preparative purposes. A260/280 ratios between 1.8 and 2.0 and the absence of contaminating elements like polysaccharides and phenols are indicators of good-quality DNA. High molecular weight fragments also indicate high-quality DNA^6^. Major factors that ensure the effective extraction of nucleic acid can be listed as cell disruption; degradation of membrane complexes and enzyme inactivation; contaminant-free nucleic acids that don’t contain any other nucleic acids, proteins, carbohydrates, or lipids; and the quantity and quality of nucleic acid^7^.

DNA extraction kits are more expensive and dependent on commercial availability, which may not be available to all researchers, or in environments with low resources, they were not included in this study. Manual methods, such as DMSO, GHCL, and Phenol-chloroform, were chosen for their reduced cost and the flexibility they offer in terms of modifying specific samples. These techniques offer a good cost-benefit ratio because they are less expensive than commercial kits without compromising DNA extraction efficiency, which makes them an appropriate choice for a variety of research environments^8^. One key benefit of manual methods is their flexibility, as they can be easily adapted to suit a wide variety of sample types and experimental requirements. Unlike commercial kits, which often have specific protocols tailored to certain sample matrices, manual methods allow researchers to modify reagents, incubation times, and processing steps to optimize the extraction process for unique or challenging samples. Additionally, manual methods are generally more cost-effective, particularly in large-scale or resource-limited settings. Commercial kits can be expensive, especially when processing large numbers of samples, while manual methods require fewer consumables and can be scaled up with minimal additional costs^9^.Conventional DNA extraction methods include Phenol-Chloroform Extraction, Guanidium Hydrochloride, Alkaline Extraction Method for plasmid DNA and *E. coli* genome, and CTAB Extraction Method for plant samples^10^. Although there are numerous ways to extract nucleic acids from various body fluids and tissues, new techniques for isolation that allow for higher yields and DNA purity are actively desired^11^. Using a pH-sensitive dye called xylenol orange (XO) and triplex reverse transcription loop-mediated isothermal amplification (tRT-LAMP), a quick and efficient nucleic acid detection method for Yellow Head Virus (YHV) in shrimp is reported. High sensitivity is attained by this colorimetric, field-deployable method, which can detect as little as 100 fg of RNA and 10 copies of the in vitro RNA transcript^12^.

One of the top global issues in food safety is preventing the spread of infectious diseases in livestock farming or aquaculture^13^. Rapid, sensitive, and cost-effective diagnostics are essential for restricting transmission and controlling disease outbreaks due to the significant challenges posed by developing infectious diseases and the rise in production demand^2^. The Pacific white shrimp is the most productive aqua-cultured crustacean species, with an estimated annual value of USD 15 billion^14^. Unfortunately, outbreaks of infectious diseases frequently affect shrimp farms, leading to the death of shrimp that have been raised and reducing both domestic and global production^15^.

Thus, new approaches that are quick, cheap, and highly sensitive are urgently needed, especially in emerging and poor countries where aquaculture is expanding quickly but the infrastructure is lacking^16^. This study proposes to determine the most efficient technique for isolating DNA from WSSV samples by comparing the sensitivity and efficiency of three distinct DNA extraction techniques: GHCL, DMSO, and phenol-chloroform.

## Materials and methods

### Disease-suspected shrimp sample collection and maintenance

A total of 30 shrimps (*Litopennaeus vannamei*) were collected from the northeast corner of Chennai, Tamil Nadu. Shrimp were observed to exhibit symptoms like lethargy and reddish discoloration, which were associated with WSSV (White Spot Syndrome Virus) infection. Animals were transferred to the laboratory from a shrimp farm with continuous aeration. The shrimp were reared in ponds with potentially altered water quality due to the presence of the virus, requiring careful monitoring^17^. The entire sampling procedure was conducted with careful consideration of ethical considerations. The appropriate authorities granted all required permissions for the shrimp’s handling and collection to guarantee adherence to environmental and ethical standards. Muscle tissue was selected for WSSV detection in this study instead of the typical target organs such as hematopoietic tissue, gills, stomach, and pleopods due to its unique advantages in terms of practicality, accessibility, and viral load. While other tissues are commonly targeted for WSSV detection, they often require more invasive procedures for sampling, which can be difficult in field conditions or on a large scale. Muscle tissue, however, is both more accessible and easier to collect, making it a more practical choice for widespread monitoring.

### Procedure

DNA extraction was performed using a lysis buffer (6%SDS, 3mM MgCl2, 15mMTri-HCL, 0.5%DMSO, 6%Acetone). DMSO is added to the lysis buffer to disrupt cell membranes and denature proteins, which facilitates the efficient release of DNA. It stabilizes the extracted DNA, preventing degradation, and improves the solubility of hydrophobic molecules. As a result, it works particularly well for extracting DNA from tissues that are difficult to lyse or from samples with resilient cellular structures. To eliminate contaminants and impurities from the DNA preparation, acetone is added to the precipitation buffer. Being a potent solvent, it aids in the dehydration of proteins and biological detritus, resulting in a DNA precipitate that is cleaner. This enhances the extracted DNA’s quality and purity, preparing it for use in PCR or sequencing.

Briefly, muscle tissue was placed into a 1.5 ml centrifuge tube and homogenized with 200 µl of lysis buffer to properly grind the tissue sample, then made up to 1 ml. Incubate this mixture at 65 degrees Celsius in a water bath for 20 min. Cool down the mixture to room temperature and add 450 µl of protein precipitation buffer (9.35 M NH4Cl, 1.15 M NaCl, and 38% ethanol). Vortex and centrifuge the mixture at 16,000 g or 11,986 RPM for 5 min at 10 degrees Celsius. Transfer the supernatant to a new tube and add 600 µl of 100% isopropanol. Centrifuge the mixture at 10,000 g or 9466 RPM for 5 min at 10 degrees Celsius. Remove the supernatant, and precipitate the DNA with 70% ethanol before resuspending it in 50 µl of nuclease-free water. Finally, the same muscle samples were extracted using a classic method, based on Guanidium hydrochloride extraction and Phenol chloroform extraction modified according to (Sobrino & Carracedo, 2013 and Montgomery & Sise, 1990).

### Determination of DNA concentration and purity

In this study, the concentration, purity, and absorbance ratios of 10 shrimp DNA samples were pooled together for each extraction method and assessed using a Thermo Scientific NanoDrop™ 2000 Spectrophotometer (ThermoFisher Scientific, USA). Each extraction method was repeated three times (*n* = 3) to ensure the reliability and consistency of results. Table 1 presents the data representing the average values from these triplicate assays. For each sample, 1 µL was pipetted onto the measurement pedestal of the NanoDrop™ instrument. To evaluate the purity of the samples, the absorbance ratios of A260/A280 and A260/A230 were calculated. The absorbance spectra were obtained for each sample within the specified wavelength range, ensuring that accurate and comprehensive data were collected for subsequent analysis^18^.

**Table 1:** DNA concentration, purity, and absorbance Ratios. Average DNA concentrations of shrimp samples, as measured using the thermo scientific nanodrop™ 2000 spectrophotometer. Each extraction method was performed in triplicate (*n* = 3).

Methods	ng/µl	260/280	260/230
GHCL	151.2	1.8	1.78
Phenol chloroform	267.8	2.0	1.93
DMSO	378.4	1.88	2.1

### PCR assay

**Fig. 1:**
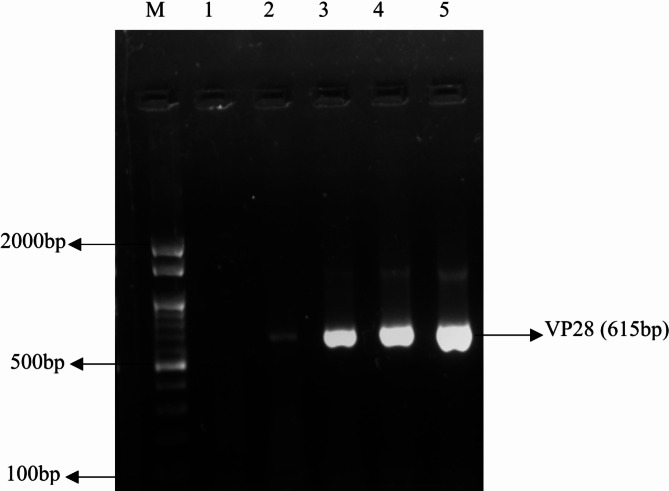
Agarose gel electrophoresis (0.8%) for extracted DNA from three different DNA extraction Methods, M-Marker (100 bp), Lane 1-Healthy shrimp, Lane 2-Positive(WSSV-Infected), Lane 3-Guainidium Hydrochloride, Lane 4-Phenol-Chloroform, Lane 5-Dimethyl Sulfoxide.

PCR analyses were performed to determine whether the DNA extracts were suitable for amplification (Fig. 1). A 1 µg/µL - DNA solution was diluted 10-fold to determine the limit of detection (LOD), or lowest order of detection, for various matrices. Serial dilutions of standard and unknown DNA were used to perform the comparative analysis. Primers used for the analysis were designed using the Primer 3 tool listed in (Table 2).

**Table 2:** Primer sequences, annealing temperatures, and amplicon length. GenBank accession No- AF440570.1.

Primers	Sequence details	GC%	annealing temp (°C)	Size (bp)
VP28 F	5’ ATGGATCTTTCTTTCACTCTTTC 3’	36.67%	57 °C	615 bp
VP28 R	5’ TTACTCGGTCTCAGTGCCAG 3’	56.67%	57 °C	
RVP28 F	5′-AGGTGTGGAACAACACATCAAG-3	55.25%	59 °C	150 bp
RVP28 R	5′-TGCCAACTTCATCCTCATCA-3′	55.25%	59 °C	

### Agarose gel electrophoresis

0.8% agarose gel (SeaKem LE agarose, Cambrex) was used for the agarose gel electrophoresis analysis of genomic DNA and amplified DNA gels)^19^. Electrophoresis used a Tris-Borate EDTA (TBE) buffer for the experiment with a 1 g/ml concentration of ethidium bromide (EtBr) at an 80 V continuous voltage for 30 min. The DNA bands were visualized and images were acquired using Gel Doc XR + Imaging system (Axygen)^20^.

### Determination of copy numbers using Real-time PCR

This study measured copy number variation (CNV) in genomic DNA using quantitative PCR (qPCR)^21^. The reaction mixture for copy number analysis was prepared by combining 1–100 ng of genomic DNA with 0.1–0.5 µM of both forward and reverse primers specific to the target gene and for SYBR Green detection, 5µL of the dye was included. Nuclease-free water was added to achieve a final reaction volume of 20 µL. The samples were then subjected to thermal cycling for amplification and fluorescence detection. Throughout the amplification process, the fluorescence that the SYBR Green dye emitted was continually measured, and the quantification cycle (Cq) value was noted. The Cq value, which is inversely related to the initial DNA quantity—the higher the DNA concentration, the sooner the Cq value—denotes the cycle at which fluorescence is above a specified threshold. The pGEMT-VP28 plasmid was serially diluted and used as a standard for the qPCR test to generate a standard curve that quantified the copy number of the target gene. The target gene copy number in unknown samples can be precisely determined which has a known and stable copy number^22^. The Cq values of the unknown samples were correlated with their corresponding copy numbers using the standard curve, which depicts the Cq value on the y-axis and the log starting quantity (in plasmid copies) on the x-axis^23^.

## Results

The phenol-chloroform and GHCL method was originally used to extract DNA from animal tissue samples. While all currently available DNA extraction techniques have proven successful in separating DNA suitable for restriction digestion or PCR amplification, they necessitate lengthy incubations, and several precipitation steps, to yield highly pure genomic DNA. The DMSO method was assessed to check its purity, quantity, and sensitivity to compare with the other two methods.

### DNA quality and quantity assessments

For both DNA concentration and purity ratios, we statistically compare the variations in yield and purity of DNA among the three extraction techniques.

The DMSO approach produced a much greater yield (378.4 ng/µL) for DNA concentration than the GHCL (151.2 ng/µL) and phenol-chloroform (267.8 ng/µL) methods. While the difference between phenol-chloroform and GHCL (*p* = 0.23) was not significant, the difference between DMSO and both phenol-chloroform (*p* = 0.01) and GHCL (*p* = 0.003) approaches was statistically significant, according to the results of the post-hoc Tukey’s HSD test. Because the DMSO approach can better extract DNA from the sample matrix without causing excessive contamination, these results show that it was more effective in terms of DNA yield (Table 1). A variety of sample matrix components, including lipids, polysaccharides, polyphenols, and extraction substances, can significantly impact the purity of DNA. The GHCL method produced DNA samples with purity ratios of 1.8 whereas the purity ratio of samples extracted by Phenol-Chloroform was 2.0. For the DMSO method purity ratios were 1.88.

### Sensitivity analysis using polymerase chain reaction

**Fig. 2:**
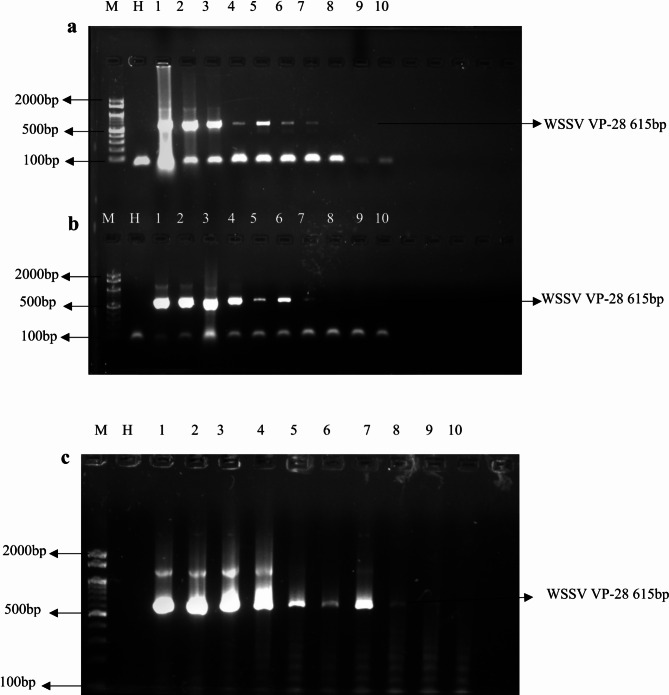
Sensitivity assay for PCR using three different DNA extraction methods. (**a**) Phenol-Chloroform extraction, (**b**) Guanidium Hydrochloride extraction, and (**c**) Dimethyl Sulfoxide extraction. The images show the detection of target DNA at various dilutions, ranging from 10⁻¹ to 10⁻¹⁰. Lane 1 represents the highest dilution (10⁻¹), and Lane 10 represents the lowest dilution (10⁻¹⁰). “M” is the molecular weight marker, and “H” represents the healthy shrimp DNA sample used as a control.

To evaluate the detection limitations of the Phenol-Chloroform, GHCL, and DMSO extraction procedures, statistical results were compared to assess the sensitivity of DNA isolated from WSSV-infected tissue. The DMSO extraction method was significantly more sensitive than the Phenol-Chloroform and GHCL procedures, which only detected up to 10^− 7^, while the DMSO method could identify WSSV DNA at a dilution of 10^− 9^ (Fig. 2). The DMSO approach is more effective at extracting DNA suitable for PCR amplification at lower concentrations, as indicated by the post-hoc Tukey’s HSD test, which confirmed that the DMSO method’s detection limit of 10^− 9^ was significantly lower than both the Phenol-Chloroform (*p* = 0.001) and GHCL methods (*p* = 0.004). Although the sensitivity of the GHCL and Phenol-Chloroform procedures is comparable, the DMSO method is more sensitive and provides a lower detection limit for WSSV DNA amplification.

### Copy numbers assessment using Real-time PCR

**Fig. 3:**
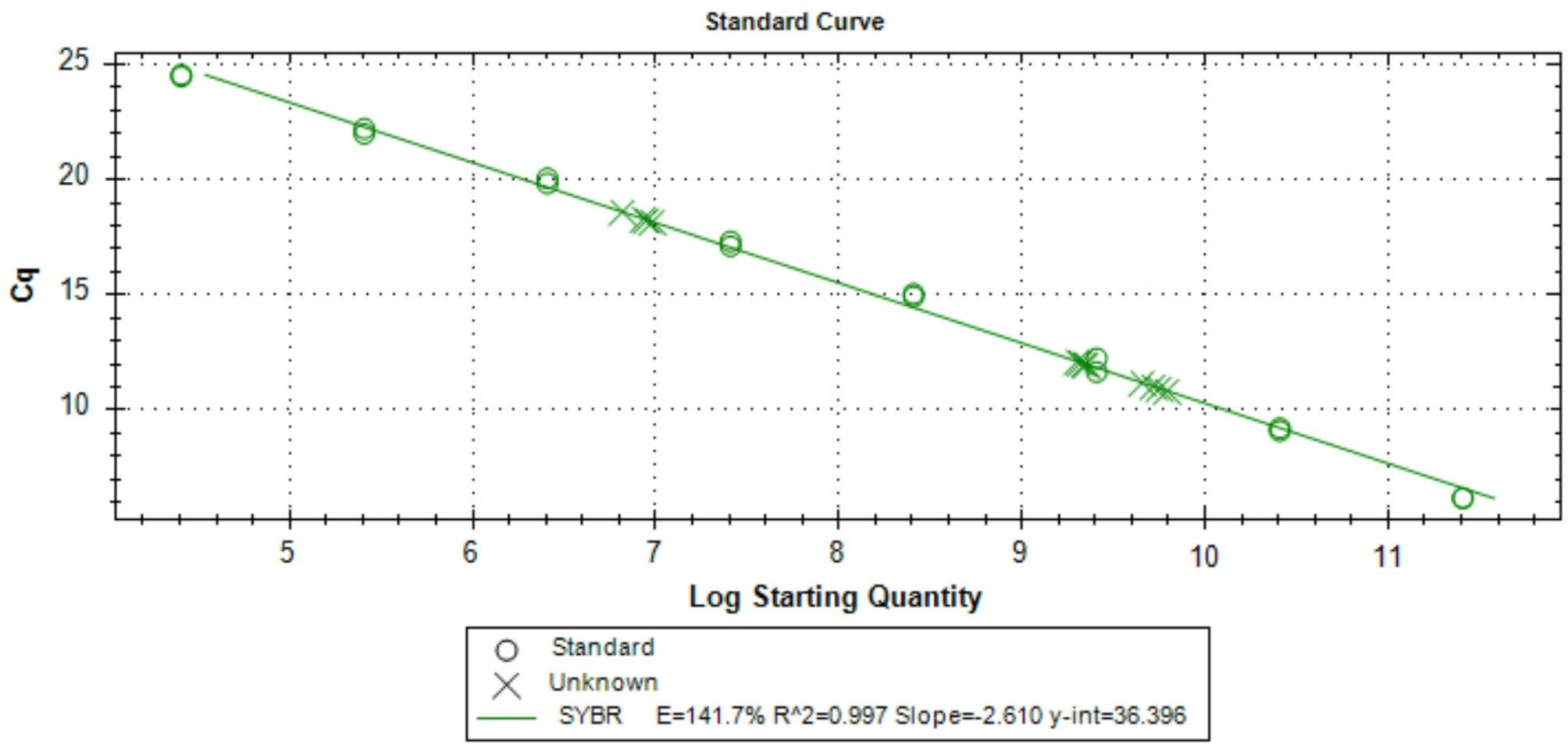
Real-time PCR amplification of serially diluted pGEMT-VP28 plasmid to determine the copy numbers of unknown samples using three different DNA extraction methods. Serial dilutions of the pGEMT-VP28 plasmid were used to create a standard curve, with the y-axis showing the Cq value (quantification cycle) and the x-axis showing the log beginning quantity (in copies of the plasmid per reaction). Plasmid dilutions ranging from 10⁻¹ to 10⁻¹⁰ copies per reaction were used to generate the standard curve. The standard curve allows for the determination of the copy number of unknown DNA samples extracted using three different methods (a) Phenol-Chloroform extraction, (b) Guanidium Hydrochloride extraction, and (c) Dimethyl Sulfoxide extraction.

Real-time copy number detection involved amplifying a test locus with an unknown copy number alongside a reference locus with a known copy number during PCR. The copy numbers for the unknown samples were calculated by comparing them to standard samples to evaluate the efficiency of DNA extraction methods (Fig. 3). The results revealed a significant difference in the copy numbers obtained between the DMSO method and the other two methods. Specifically, the DMSO method achieved a notably higher copy number (4.89E + 09) compared to the GHCL (4.16E + 07) and Phenol-Chloroform (7.78E + 06) methods (Table 3). A post-hoc Tukey’s HSD test confirmed that the DMSO method produced significantly higher copy numbers than both the GHCL (*p* < 0.001) and Phenol-Chloroform methods (*p* = 0.002). The increased copy number observed with the DMSO method may indicate superior DNA recovery and reduced degradation during extraction. In contrast, the Phenol-Chloroform and GHCL methods yielded lower copy numbers, potentially due to inefficiencies in DNA isolation or loss during purification steps. These findings suggest that DMSO provides a more effective approach for extracting viral DNA, ensuring higher quality and yield of DNA for downstream analysis applications.

**Table 3:** Three different DNA extraction methods and their copy numbers.

Methods	Copy Numbers
Guanidium Hydrochloride Method	4.16E + 07
Phenol-Chloroform Method	7.78E + 06
Dimethyl Sulfoxide (DMSO) Method	4.89E + 09

## Discussion

Since it has been proposed that DNA extraction is a crucial step in the functioning of molecular DNA technologies, including PCR-based techniques, researchers have employed several modified processes to extract DNA from samples of animal cells effectively. To compare the quality and quantity of the extracted nucleic acid from various DNA extraction techniques two protocols (Phenol-Chloroform/GHCL) and one new protocol using in-house reagents were assessed. A preliminary assessment of the quality of the DNA was carried out using 1% agarose gel electrophoresis. The high molecular weight genomic DNA band is only visible on the gel using all techniques. The yield and quality of the isolated DNA were also confirmed by spectrophotometric analysis. Specifically, we found that the DMSO approach produces high-quality DNA in multiple samples. The quality of the DNA produced by the other methods is medium-quality. The A260/A230 ratios were also established to assess the amounts of contamination caused by the presence of salts, peptides, and polysaccharides.

One of the primary reasons for DMSO’s success is its ability to efficiently lyse shrimp tissues, which are often tough and rich in chitin. DMSO is a powerful solvent that breaks down cellular structures and denatures proteins, including nucleases, which is particularly critical when dealing with shrimp tissues that are high in enzymatic activity which could otherwise degrade the DNA during extraction. This results in a high yield of intact DNA^24^. While the phenol-chloroform extraction method is widely used, it can be less efficient for shrimp tissue, which contains a large amount of protein and chitin. This method involves multiple organic solvent phases, which can lead to incomplete tissue lysis and potential degradation of the DNA^25^. Similarly, the GHCL-based method, while effective for many organisms, may not efficiently address the unique challenges posed by shrimp tissue, such as the presence of chitin, which can hinder extraction. Chitin, a tough polysaccharide, can impede the efficient breakdown of tissue and release of DNA. DMSO is particularly well-suited for breaking down this chitinous barrier, allowing for better access to the DNA inside cells. This makes DMSO a more reliable option when working with shrimp tissue, which requires efficient lysis to extract high-quality genetic material. DMSO also minimizes the contamination of the extracted DNA, an important consideration when working with any biological sample. The use of phenol-chloroform, on the other hand, can result in contamination with phenolic compounds, which may interfere with downstream applications like PCR or sequencing. Although the GHCL-based method is effective in many cases, it does not always remove contaminants as efficiently as DMSO, and the DNA may still contain chaotropic salts that could hinder further molecular analyses. DMSO-based DNA extraction is simpler, safer, and more efficient than phenol-chloroform and GHCL methods. Unlike phenol-chloroform, which involves toxic chemicals requiring careful handling, DMSO is a safer alternative, reducing the risk and simplifying the process. GHCL methods, while safer, often require complex steps to remove contaminants and may yield inconsistent results with shrimp tissue^26^. DMSO also consistently produces high-quality genomic DNA, which remains intact and free from degradation, crucial for downstream applications like PCR and sequencing. Additionally, DMSO-based extraction is more time- and cost-efficient. Although there may be concerns about reagent availability, DMSO is a widely used and cost-effective option reagent in molecular biology and its stable supply is guaranteed by several sources. Aquaculture businesses of all kinds, from small farms to huge commercial facilities, can benefit from its ease of scalability and storage. These elements ensure that DMSO continues to be a dependable and useful option for routine diagnostics in aquaculture by reducing the likelihood of logistical issues. To preserve DNA integrity over time, DMSO-treated samples might need to be stored under specific circumstances, such as at -20 °C or below.

Due to its flexibility, DMSO (dimethyl sulfoxide) is widely used in molecular biology. By inhibiting ice crystal formation, it preserves cells and tissues during freezing^27^. Additionally, DMSO serves effectively as a solvent for preparing reagents and dissolving hydrophobic substances^28^. It enhances gene transfection by increasing the permeability of cell membranes, allowing DNA to enter cells more easily. Moreover, it can induce cell differentiation, especially in studies involving stem cells^29^. In biochemical tests, DMSO is also utilized to inhibit enzyme activity^30^. Therefore, DMSO plays an essential role in advancing biological and biochemical research.

DNA extraction kits were not included in the comparison with the novel DNA extraction technique for shrimp virus DNA extraction due to their high cost. Commercially available DNA extraction kits, although convenient and widely used in molecular biology, can be prohibitively expensive, especially for large-scale studies or routine applications^31^. The cost of these kits could significantly increase the overall expenses of the research, limiting their accessibility for many laboratories, particularly in resource-constrained settings^32^.

There have been many attempts in the past few decades to develop a quick, safe, low-cost, and highly effective methodology for high-quality DNA extraction. Methods capable of eliminating the various amplification reaction inhibitor chemicals are necessary for the first crucial stage in molecular analytical techniques, which is DNA extraction, purification, and concentration. Samadi Shams et al. described a highly successful DNA extraction method from fresh, frozen, dried, and clotted blood samples. However, this approach uses cetyltrimethylammonium bromide (CTAB), which lowers the amount of recovered DNA that is ultimately retrieved. Without liquid nitrogen or phenol^33^, have published the DNA extraction procedure for plants with high quantities of polysaccharides and secondary compounds. The most effective technique for extracting DNA from shrimp was the DMSO approach, which was shown to have excellent DNA yields, a low acquisition curve, lower costs than commercial kits, and the ability to extract DNA at high throughput.

The safety of laboratory personnel and environmental sustainability are key considerations when selecting DNA extraction methods for large-scale applications. As noted in previous studies, the use of toxic chemicals such as phenol and chloroform poses significant health risks. It requires careful disposal, adding to the environmental footprint of these methods. Disposing of toxic reagents such as phenol, chloroform, and GHCL presents significant environmental challenges. Phenol and chloroform are classified as hazardous waste, and improper disposal can lead to contamination of water supplies and soil, which poses risks to both terrestrial and aquatic ecosystems^34^. In addition to the environmental risks, the disposal of these chemicals requires costly and time-consuming procedures to ensure compliance with safety regulations. This creates an additional burden on laboratories and research facilities, further emphasizing the need for safer and more environmentally friendly DNA extraction methods. Similarly, GHCL presents safety challenges due to its corrosive and toxic properties. In contrast, DMSO is a safer and more environmentally friendly reagent that can be handled with fewer precautions, and its toxicity profile is much lower compared to phenol and GHCL. This not only reduces the risk of exposure to laboratory workers but also contributes to a more sustainable approach in aquaculture diagnostics, aligning with current efforts in the scientific community to minimize the environmental impact of research practices. Furthermore, previous research has indicated that Guanidium Hydrochloride (GHCL), despite being highly effective in extracting DNA from low-copy pathogens, can lead to degraded DNA in some cases, especially if not handled with precision. In contrast, DMSO maintains DNA integrity without the need for stringent handling or toxic reagents, ensuring a higher success rate and consistent DNA quality, even with challenging tissue samples^35^.

By offering a safer, more efficient, and affordable alternative, the DMSO-based method holds great promise for large-scale diagnostics in aquaculture, particularly for the detection of White Spot Syndrome Virus (WSSV) in shrimp, and may have broader applications for other aquatic pathogens.

## Conclusion

Biomolecule extraction techniques, such as guanidinium hydrochloride, and phenol-chloroform extraction, which is frequently used in DNA extraction, have assisted scientists and researchers in manipulating subsequent molecular biology analysis to have a better understanding of the biological materials of the earth. In the study given here, we developed a quick, low-cost, and effective methodology for extracting high-yield genomic DNA from shrimp. Three different DNA extraction techniques were tested in this study to extract high-quality DNA that can be effectively amplified using PCR. For routine aquaculture diagnostics, the DMSO approach has several benefits, especially because of its high DNA yield. DMSO is perfect for small or deteriorated samples that are frequently seen in aquaculture because it effectively breaks down cell membranes, allowing more DNA to be released. The method’s cost-effectiveness and quickness are two other important benefits. The DMSO approach is faster and less expensive than other techniques, such as phenol-chloroform, and requires fewer steps and chemicals, which makes it appropriate for frequent usage in aquaculture diagnostics. This is particularly advantageous for procedures that call for quick genetic screening or disease identification. Therefore, the DMSO method’s simplicity, safety, and cost-effectiveness make it a more attractive option for researchers working with shrimp and other similar organisms. In conclusion, it provides superior efficiency in tissue lysis, better handling of chitin, minimizes contamination, and results in high-quality DNA suitable for molecular analysis.

## Data Availability

All authors are sure that all data and materials as well as the software applications support their published claims and comply with the field standards.

## Abbreviations

WSSV: White spot syndrome virus
GHCL: Guanidium hydrochloride
DMSO: Dimethyl sulfoxide
PCR: Polymerase chain reaction
EtBr: Ethidium bromide
TBE: Tris-Borate Ethylenediaminetetraacetic acid
